# UNAIDS ‘multiple sexual partners’ core indicator: promoting sexual networks to reduce potential biases

**DOI:** 10.3402/gha.v7.23103

**Published:** 2014-03-11

**Authors:** Zacharie Tsala Dimbuene, Jacques B.O. Emina, Osman Sankoh

**Affiliations:** 1 PRONUSTIC Research Laboratory, Department of Demography, University of Montreal, Montreal, Quebec, Canada; 2 Faculté des sciences économiques et de gestion, Département des Sciences de la population et du développement, Université de Kinshasa, Kinshasa, Democratic Republic of the Congo; 3 Research Data Centre Program, Microdata Access Division, Statistics Canada, Ottawa, ON, Canada; 4 Department of Sociology and Anthropology, University of Ottawa, Ottawa, ON, Canada; 5 INDEPTH-Network, East Legon, Accra, Ghana; 6 CEPS/INSTEAD, University of Luxembourg, Walferdange, Luxembourg; 7 School of Public Health, University of the Witwatersrand, Johannesburg, South Africa; 8 Faculty of Public Health, Hanoi Medical University, Hanoi, Viet Nam

**Keywords:** HIV/AIDS, risky sexual behavior, health demographic and surveillance systems, sexual networks

## Abstract

UNAIDS proposed a set of core indicators for monitoring changes in the worldwide AIDS epidemic. This paper explores the validity and effectiveness of the ‘multiple sexual partners’ core indicator, which is only partially captured with current available data. The paper also suggests an innovative approach for collecting more informative data that can be used to provide an accurate measure of the UNAIDS’s ‘multiple sexual partners’ core indicator. Specifically, the paper addresses three major limitations associated with the indicator when it is measured with respondents’ sexual behaviors. First, the indicator assumes that a person’s risk of contracting HIV/AIDS/STIs is merely a function of his/her own sexual behavior. Second, the indicator does not account for a partner’s sexual history, which is very important in assessing an individual’s risk level. Finally, the 12-month period used to define a person’s risks can be misleading, especially because HIV/AIDS theoretically has a period of latency longer than a year. The paper concludes that, programmatically, improvements in data collection are a top priority for reducing the observed bias in the ‘multiple sexual partners’ core indicator.

Over the past three decades, risky sexual behavior (RSB) has been one of the most documented topics in HIV-related studies to further our understanding about the probability of an individual being exposed to an infected person. Hence, RSB has driven most HIV interventions and programs worldwide – especially in sub-Saharan Africa (SSA), which is the most affected region. Scholars and policymakers define people engaging in higher RSB as those who have: 1) sexual activities that involve passage of bodily fluids, 2) sexual intercourse without the use of a condom, and/or 3) multiple sexual partners (e.g. serial sexual monogamy or concurrent sexual partnerships) (1).

Identifying people who engage in RSB from respondents’ self-reported number of sexual partners is the widely used approach for studying the determinants of sexually transmitted infections (STIs)/AIDS; however, this approach has major limitations. First, the UNAIDS core indicator posits that an individual’s risk of contracting HIV/AIDS/STIs are a function of his/her own sexual behavior. Such a definition precludes a partner’s sexual history, which can be the key determinant of an individual’s risk of contracting HIV. Second, the cross-sectional nature of data and the short 12-month time span considered can be misleading, especially in the case of HIV/AIDS because of its long incubatory period. Although informative, recent RSB during the past 12 months provides little evidence for improving the effectiveness of reproductive health (RH) interventions and programs. For instance, sexual monogamy cannot be viewed as a fully protective factor when a partner’s sexual history, sexual networks, and STIs/HIV status are ignored or when consistent condom use is undocumented. Likewise, multiple sexual partners are not a risk factor per se; rather, the consistent use of condom will determine more accurately the levels of one’s risks. Findings from the Africa Centre Demographic Surveillance site in KwaZulu-Natal, South Africa, revealed that, in the immediate local community, the mean lifetime number of male partners was predictive of the risk of contracting HIV among women. In contrast, a high prevalence of sexual partnership concurrency within the local community was not associated with the likelihood of contracting HIV (2).

Against this background, this study explores the limitations of current measures of multiple sexual partnerships with a sharp focus on measurement and data collection, and it discusses the potential biases. Furthermore, the paper suggests a method for gathering more reliable data through existing AIDS surveillance in order to provide new insights for successful RH policies and HIV/AIDS interventions.

## Risky sexual behavior: data collection and HIV risk assessment

UNAIDS’s indicators for monitoring HIV risk in general populations are widely documented (1–4) and are presented in Table 1. These indicators are defined in a number of ways using respondents’ sexual behavior. The most widely used operational definition of RSB is based on the behavior itself; it encompasses early sexual activity and unprotected sexual activity, the number and types of sexual partners, relationship to partner, frequency of sexual intercourse, and condom use. A second but neglected way refers to the nature of the partner: if a partner is HIV-positive, whether a partner is an intravenous drug user, or if a partner is nonexclusive.

**Table 1:** UNAIDS current indicators

Indicator	Name	Denominator	Numerator
All adults			
UN1	Higher risk in the last year	All who had sex in the last year	People who had sex with non-cohabiting partner in last year
UN2	Condom use at last higher risk sex	All who had sex with non-cohabiting partner in last year	People who used a condom at most recent sex with non-cohabiting partner
UN3	Commercial sex in last year	All men	Men who had sex with commercial sex worker in last year
High-risk groups			
UN4	Condom use at most recent commercial sex (client report)	Men who reported commercial sex in the last year	Men who used condoms in most commercial sex
UN5	Condom use at most recent commercial sex (sex worker report)	Sex workers who have had sex with a client in the last year	Sex workers who used a condom when they had sex with their most recent client
UN6	Higher risk male–male sex in the last year	All men in a special survey of men who have sex with men	Men who had anal sex with at least one man in the past 6 months
UN7	Condom use at most recent anal sex between men	Men who have had anal sex with a man in the past 6 months	Men who used a condom at the most recent occasion they had anal sex with a man
Those aged 15–24 years			
UNy1	Median age at first sex among young women and men	The age at which 50% of young people aged 15–24 say they have already had sex	
UNy2	Young people having premarital sex in the last year	All young people who have never had a cohabiting partner	Young people who have never had a cohabiting partner
UNy3	Young people using a condom during premarital sex	All young sexually active people who have never had a cohabiting partner	Those who used a condom at their most recent sex
UNy4	Young people having multiple sexual partners in the last year	All young people	Young people who report more than one sexual partner in the last year
UNy5	Young people using a condom at the last higher-risk sex	All young people	Young people who used a condom at the most recent sex with a non-cohabiting partner in the last year
UNy6	Condom use at first sex	All young people who have ever had sex	Young people who used a condom the first time they had sex
UNy7	Age mixing in sexual relationships	Women aged 15–19 who had sex in the past 12 months with a man to whom they are not married	Women aged 15–19 who had sex with a man to whom they are not married and who is 10 or more years older (based on their last three reported partnerships)

Source: Adapted from Slaymaker (4).

## Data collection: current approaches

There are two general approaches used to collect data about sexual partnerships in the general population (5). The first asks respondents if he/she has had additional sexual partners during a specific partnership in the last 12 months preceding the survey. It is obvious that if characteristics of the partners are not collected, it is difficult to determine the epidemiological importance because some partnerships may be one-time sexual encounters. A calendar method, in which detailed information about sexual partnerships – including the start and end dates of sexual relationships and the characteristics of the partners (e.g. regular, causal, commercial) – allows researchers to create sexual partnership calendars and to construct various definitions of concurrency. This method provides rich data; however, there may be recall bias especially pertaining to the start and end dates of partnerships. These two approaches are limited due to social desirability and self-reporting biases; nevertheless, they provide useful data to compute UNAIDS core indicators.

**Fig. 1:**
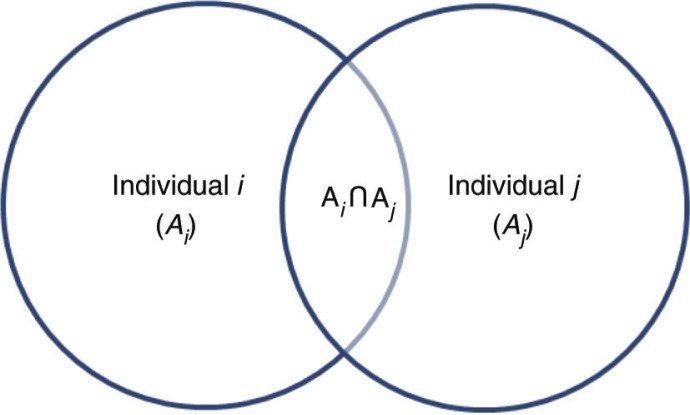
Risk contribution for sexual partners.

Notwithstanding the progress in monitoring AIDS around the world, the aforementioned approaches still remain *respondent-oriented* because information is only collected from selected respondents. Although the calendar method collects information about sexual partnerships (e.g. partners’ characteristics: age, type of partner, or relationship) and condom utilization with each sexual partner, it does not collect information about a partner’s sexual history. The current study argues that this information is very important to accurately evaluate a person’s HIV risk. For example, suppose two individuals (*i, j*) are in a sexual partnership in which only individual *i* was randomly selected and interviewed, and for the number of sexual partners (*n*_*i*_ *, n*_*j*_) for individuals *i* and *j*, *n*_*i*_ equals 1. If RSB is defined as a binary variable coded 0 (or ‘no risk’) when *n*_*i*_ *<*2 and 1 (‘at risk’) when *n*_*i*_ *≥*2, then individual *i* is not at risk at all. This sounds logical but, practically, the true risk associated with an individual’s sexual behavior is contingent upon the partner’s sexual behavior *j*. Now suppose that individual *j* had many sexual partners (i.e. *n*_*j*_≥2). If, by any chance, one of his/her sexual partners is infected, the individual *i* is then ‘at-risk’ of contracting AIDS. The current definition and computation for RSBs associated with sexual multipartnership (see Fig. 1) supposes that *i*’s and *j*’s sexual behaviors contributing to the likelihood of contracting an STI are independent (i.e. *A*_*i*_ *∩ A*_*j*_*=φ*), where *A*_*i*_ and *A*_*j*_ are the sexual behaviors of individuals *i* and *j*, respectively. Furthermore, *P(A*_*j*_ *)*=0, concluding that only individual *i* contributes to the theoretical risk of contracting an STI. This is not necessary true in the real world.

Because the risks referred to here are not independent, the Bayes’ theorem can be used to estimate the bias when researchers and practitioners merely ignore a partner’s sexual behavior:

1 $$P\left(A_{i}|A_{j}\right)=\frac{P\left(A_{j}|A_{i}\right)\text{*}P\left(A_{j}\right)}{P\left(A_{j}\right)}$$

From eq. (1), it can be seen that the contribution of individual *j* is a key element in determining the ‘true’ level of the RSB indicator because the risks of contracting HIV/AIDs are not independent. Furthermore, *P(A_j_)* cannot be zero because people engaging in sexual activities (e.g. non-virgins) are at risk for contracting STIs/HIV/AIDS; one’s risk can only be minimized. Whichever level of the contribution of the individual *j* that is considered, the RSB indicator is likely biased if *j*’s sexual behavior is ignored. The level of the RSB indicator is overestimated when *P(A_j_)*→ 0, and it is underestimated when *P(A_j_)*→ 1. The practical question is under what conditions would the indicator be considered under- or overestimated? Because *j*’s sexual behavior is not known, researchers and policymakers cannot accurately determine the levels of the UNAIDS core indicator in the general population or which segments of the population are at a higher risk of HIV/AIDS/STIs.

## The length of the relationship: another key factor of mismeasurement of UNAIDS “multiple sexual partners” core indicator

In addition to ignoring the partner’s sexual behavior, RSB indicators are often based on a very short period of time (e.g. 12 months). This assumption holds when the period of latency is short as in the case of STIs (e.g. chlamydia, gonorrhea). Not only might a person’s sexual behavior vary considerably within this short time period but this time span can also be misleading in the case of AIDS because it does not account for AIDS’ long period of incubation.

Let us assume that the 12-month period is sufficient for providing reliable RSB core indicators. Additionally, let us assume that the individuals (*i, j*) have been faithful in their relationship (i.e. *n*_*i*_ *=n*_*j*_=1). Under this first assumption, individuals *i* and *j* are classified in the ‘no-risk’ group because they have only had one sexual partner during the 12 months preceding the survey. This is the ideal scenario of *sexual monogamy* that RH programs and interventions engender under the partner reduction approach.

Let us consider two additional assumptions. First, suppose that the sexual behaviors of the individuals *i* and *j* significantly differed before the one-year period and the beginning of their relationship (i.e. the time period in which the data were collected). More specifically, assume that individual *j* had many sexual partners during the year preceding the one-year survey period (i.e. *n*_*j*_ *→ ∞*). This information is key for more precisely determining the ‘true’ risk for individual *i* within this short period. However, most available data do not contain information about partners’ sexual histories. Second, assume that individual *j* is infected. Then *j*’s serostatus impacts the level of risk associated with individual *i*. By and large, these assumptions imply that individual *i* is *falsely* not ‘at-risk’ because each individual has had only one sexual partner during the last 12 months.

Current approaches, including UNAIDS’s definition of RSB and associated core indicators, have neglected partners’ sexual histories. This is an error because sexual history is crucial for determining a person’s risk of contracting HIV/AIDS. Suppose that individual *j* was committed in the current relationship but is infected with an STI/HIV/AIDS. Based on the definition of RSB used in HIV-related studies, individual *i* did not contribute to the numerator of the RSB indicator because he/she was classified in the ‘no-risk’ segment of the population; therefore, the indicators obtained with this computation are overestimated.

## Continuous sexual network studies: a window of improvement

Researchers and policymakers advocate that targeted interventions and evidence-based prevention programs are cost-effective strategies to combat STIs/HIV/AIDS. Therefore, more relevant data on RSB are of top priority. Sociocentric surveys of sexual partnerships allow identification of the population-level structural characteristics of sexual networks and the position of STI/HIV-positive persons within these networks over time. A network consists of a set of nodes representing people, connected by a set of edges representing mutual relationships. It can be partitioned into a group of individuals (components) within the network, connected either directly or indirectly through sexual partnerships (6–8). Empirically, a cross-sectional study conducted on Likoma Island, Malawi, reported that a large majority of all sexually active respondents were connected to a network component or were linked through multiple independent chains of sexual relationships (9). This approach allows the identification of the different types of sexual partnerships in which people are involved. Basically, those relationships can be as follows:

1. True monogamous partnership: people mutually reporting one partner
2. Indirect multiple partnerships: people who report one partner, although his/her partner has, in fact, many sexual partners
3. Simple multiple partnership: people reporting many sexual partners; in contrast, these later report only one sexual partner
4. Indirect concurrent partnerships: people who reported one partner, whereas his/her partner had sex between two coitus or sexual intercourse with one partner occurred between two coitus with another partner
5. Direct concurrent partnerships: people who reported overlapping sexual partnerships in which sexual intercourse with one partner occurs between two coitus with another partner
6. Complex multiple partnerships: people who reported many sexual partners who are themselves involved in many sexual relationships with other persons

The sexual networks approach is used within the sexual transmission infection surveillance data from communicable disease control to monitor sexual network dynamics over time (8). The indicator allows people at higher risk of HIV to be identified (2–4, 6, 7), especially when sexual intercourse is unprotected. However, selection biases may affect data collected from health facilities, particularly in the context of low access to health facilities – especially in SSA. In low- and middle-income countries (LMICs), a unique opportunity for better data collection from people engaging in RSB is offered by health and demographic surveillance system (HDSS) field sites of the International Network for the Demographic Evaluation of Populations and Their Health (INDEPTH). INDEPTH (www.indepth-network.org) was established in 1998. Since then, 49 HDSSs became operational in Africa, Asia, and Oceania, run by 42 health research centers. INDEPTH provides an environment in which researchers from the HDSS sites can jointly collect data and compare findings mostly through working groups built upon specific research agendas. The network also offers a unique platform covering a total population of more than 2.4 million individuals in SSA.

An HDSS consists of monitoring demographic and health characteristics of a population living in a well-defined geographical area (9, 10). The process starts with a baseline census followed by regular update of key demographic events (births, deaths, and migration) and health events. In addition to sociodemographic characteristics, a typical HDSS also includes age at first sex, number of partners, recent sexual activity, and contraceptive use. Furthermore, an HDSS provides a platform for investigating any population and health issues and for facilitating the evaluation of interventions to improve the well-being of the population (11). The implementation of network studies in an HDSS that allows classifying people based on more detailed RSB can deepen our understanding about one’s risk of contracting HIV/AIDS/STIs. Hence, this approach provides a more accurate measurement of the ‘multiple sexual partners’ core indicator using a sexual partner’s history module to collect information about the individuals with whom the respondent had sexual intercourse since the last data collection.

The interviewer should ensure respondent’s privacy before the start of the interview because of the sensitivity of the module regarding the partner’s sexual history. The conditions surrounding the respondents’ privacy should be assessed and recorded by the interviewers. In addition, respondents should be reminded to report all sexual partnerships including one-off sexual partnerships as well as sexual partnerships with sex workers (12). Questions about partners are specifically framed around sexual partners and questions about dates should refer to sexual intercourse (12). Respondents in HDSS are given a unique identifier. It is possible to link a respondent’s sexual history with his/her sexual partnerships; therefore, when this information is collected, it is possible to construct the sexual network in the demographic surveillance area (DSA). Finally, this rich information leads to the identification of the following sexual partnerships:

1. True monogamous partnership
2. Indirect multiple partnerships using condoms
3. Indirect multiple partnerships not using condoms
4. Simple multiple partnerships not using condoms
5. Simple multiple partnerships using condoms
6. Complex multiple partnerships using condoms
7. Complex multiple partnerships not using condoms

## Discussion

RSB is of paramount importance in the fight against HIV/AIDS. To assist researchers and policymakers in their work, UNAIDS developed a series of core indicators to measure RSB and to gauge changes in HIV/AIDS around the world.

There are three findings of importance. First, engaging in sexual monogamy is not fully safe when a partner’s sexual history is not taken into consideration. To better understand a person’s risk of contracting HIV/AIDS/STIs, it is recommended that sexual behaviors be analyzed within sexual network configurations. Second, most indicators, including RSB, are often based on a one-year study period. This timeframe holds when the latency period is short, such as for STIs (e.g. chlamydia, gonorrhea). The indicator associated with RSB is designed to monitor changes in the evolution of the AIDS epidemic around the world; therefore, the short period of time (12 months) is questionable except within a longitudinal frame such as an HDSS. Furthermore, having one sexual partner during the past 12 months is not truly safe. An additional criterion, the HIV-status of the sexual partner, is required. Recent AIDS programs encourage voluntary HIV testing. However, HIV testing may be effective only if committing partners agree to begin their sexual relationship after HIV testing. This is not very common in SSA. Third, the INDEPTH network of HDSS field sites is a unique platform for monitoring sexual behavior within a demographic area, especially when analyses at individual and community levels are to be performed.

Previous research on RSB has been largely based on respondents’ self-reported RSBs collected in demographic and social surveys (4). That may partly explain the lower effectiveness of many HIV interventions in SSA because reliable data are lacking. Indeed, UNAIDS’s indicators are of limited importance for implementing more effective HIV interventions. Self-RSBs have a number of methodological issues that affect the reliability and validity of the indicators. These issues range from a participant’s literacy level and comprehension of behavioral terminology to recall biases and respondents’ confidentiality. Self-reported sexual behaviors have been criticized for two main reasons: sexuality is a sensitive topic. Therefore, individuals may adapt their responses according to social desirability (‘I tell you what you want to hear’). The validity of respondents’ self-reported sexual behaviors is also a subject of debate. For instance, previous studies reported that measurement of age at premarital intercourse is considerably problematic, especially for older youths (3). Researchers have questioned the consistency and reliability of self-reported age at first sex in survey results from developing countries (13–15). Findings indicated that age at first sex varied widely by interview modes and over time (14). Likewise, analyses indicate that underreporting appears to be widespread among girls compared to boys (3).

In SSA, the social context may likely undermine the accuracy of self-reported age at first sex. For boys, having sex represents a prestige among peers, while it is shameful for females to be sexually active in societies where virginity is still rewarded. In the same vein, researchers should consider social desirability bias or the tendency of women to underreport or men to overreporting in order to conform to socially acceptable behaviors. In practice, precautions are usually taken to limit but not to eliminate the magnitude of social desirability bias during the interviews. Hence, social desirability bias may constitute a significant limitation of the UNAIDS core indicators.

## Conclusion

Three decades after the outset of HIV/AIDS, SSA still remains the most affected region in the world. Although some progress has been made regarding monitoring the pandemic, it is worthy to question the effectiveness of HIV/AIDS interventions and programs. Through the sexual networks approach, this paper advocates a major shift in HIV/AIDS data collection in order to gather more appropriate data that would provide reliable HIV/AIDS indicators for protecting people in SSA and around the world. Indisputably, this will require time and money; however, the gain will be substantial. The dynamics of sexual networks should be taken into consideration because these would allow a focus on individual and community characteristics. Previous research has shown that the structure of a contact network can have a profound effect on the dynamics of infectious diseases that are transmitted through it (16). Of course, this approach also raises methodological challenges for researchers in this field in terms of data collection (e.g. how to overcome recall bias using date-based measures to estimate concurrency) and in terms of statistical analyses of sexual networks that can be unfamiliar to researchers.
